# Acute Water Ingestion as a Treatment for Postural Orthostatic Tachycardia Syndrome

**DOI:** 10.19102/icrm.2019.100206

**Published:** 2019-02-15

**Authors:** Jeffrey B. Ziffra, Brian Olshansky

**Affiliations:** ^1^Department of Cardiology, Mercy Medical Center, Mason City, IA, USA; ^2^Department of Cardiology, University of Iowa, Iowa City, IA, USA

**Keywords:** Autonomic dysfunction, postural orthostatic tachycardia syndrome, syncope

## Abstract

A 24-year-old female presented to our clinic with symptomatic tachycardia. In the clinic, she was able to replicate her symptoms, which were due to tachycardia in a standing position that resolved upon sitting. The patient was then offered eight ounces (236.6 mL) of water and, after consumption of such, the standing tachycardia was no longer observed. A diagnosis of postural orthostatic tachycardia syndrome (POTS) was made. This case report discusses a novel approach to acute treatment for POTS.

## Introduction

Postural orthostatic tachycardia syndrome (POTS) is an orthostatic intolerance syndrome often seen in young females in which symptoms and tachycardia occur with standing. Although no treatment has yet been proven to be ideal, supine exercise and hydration may provide some benefit. We describe a case of a 24-year-old female with POTS whose standing tachycardia was eliminated after drinking eight ounces (236.6 mL) of water. The effects of water ingestion as a treatment for POTS have rarely been described.

## Case presentation

**Figure 1:**
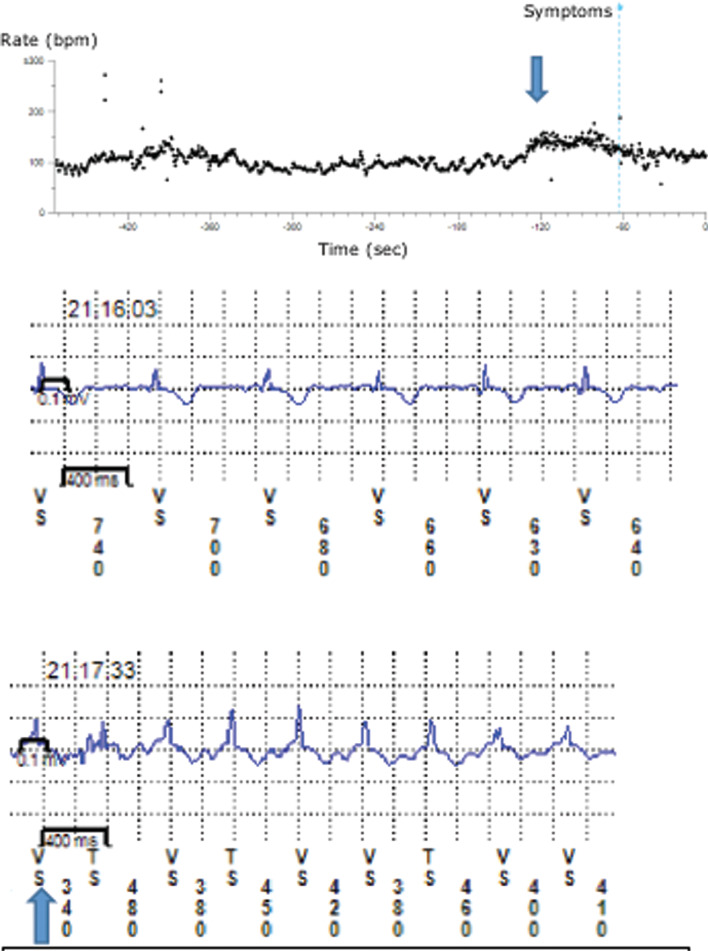
Loop recorder representative strip of a gradual increase in sinus rate.

A 24-year-old nurse with syncope and palpitations had undergone an evaluation that included loop recorder implantation and tilt-table test two years prior, with negative results. She presented to our clinic after her symptoms worsened and was noted to have multiple episodes of tachycardia recorded on her loop recorder the month before. While sitting in the clinic, she was found to be in sinus rhythm, with a heart rate of 75 beats per minute (bpm). Additionally, her resting blood pressure was 112/72 mmHg while sitting. However, during continuous monitoring via her loop recorder, within seconds of standing, sinus tachycardia (rate: 150 bpm) ensued without an appreciable drop in blood pressure **(Figure 1)**. This was reproduced multiple times during this clinic visit. She was symptomatic at this heart rate. Upon sitting, her heart rate returned promptly to 75 bpm and her symptoms resolved. This was deemed consistent with a diagnosis of POTS.

As water ingestion can help some patients with orthostatic hypotension, we thought that such might be effective in her case as well. We had her sit and drink eight ounces (236.6 mL) of water. Ten minutes later, upon standing, her heart rate remained identical to her resting heart rate. This effect was reproducible.

Acute water ingestion can elevate^1^ blood pressure acutely in patients with orthostatic hypotension, but its use as an acute treatment for POTS has not yet been widely reported, to our knowledge. Along with initial conservative measures (eg, water intake), we prescribed atenolol. The patient subsequently demonstrated resolution of her symptoms. She was followed using a Reveal LINQ™ (Medtronic, Minneapolis, MN, USA) implantable loop recorder. With more frequent and regular hydration, the tachycardia and symptoms abated.

## Discussion

POTS is a chronic, systemic orthostatic intolerance disorder characterized by a heart rate increase of at least 30 bpm (or 40 bpm in individuals aged 12–19 years) without a corresponding drop in blood pressure.^2,3^ Associated symptoms include lightheadedness, weakness, palpitations, fatigue visual changes, “brain fog,” presyncope, and exercise intolerance.^3,4^ POTS occurs most commonly in women aged between 15 years and 45 years, with an incidence rate of 0.2%.^3,5^

The pathophysiology of POTS is not fully understood, but several mechanisms have been considered. Patients with POTS have orthostatic intolerance due to the inability to adequately vasocontrict veins in the legs and/or splanchnic circulation. In one study, POTS patients were compared with healthy controls to investigate autonomic function and heart size in response to exercise. Results indicated that patients with POTS had, on average, 16% smaller hearts versus the controls. Exercise training increased ventricular cavity size and symptoms.^6^ These authors suggested that such patients with smaller hearts who have POTS should be considered to have “Grinch syndrome,” referring to the Dr. Seuss character whose heart was “two sizes too small.”^6^

A separate study by the Mayo Clinic^5^ aiming to evaluate the prevalence and pathogenetic mechanisms of POTS found that, in a population of 152 patients, at least half of the cases had a neuropathic basis and that a substantial percentage of cases could be of autoimmune origin (ie, at least one in seven patients). Potential risk factors for POTS in children and adolescents include a faster supine heart rate, less water intake, and shorter sleeping hours.^7^

Currently, there are no clinical trial– or guidelines-established treatments for POTS or class I recommendations for its therapy. Although one study has indicated that one treatment may yield better outcomes over another,^8^ another suggested a multidisciplinary approach that includes both conservative and nonpharmacologic measures may be best.^9^ Examples^5^ include withdrawing offending medications, applying waist-high compression stockings, and increasing water intake (2–3 L/day) and salt intake (10–12 g/day). Symptoms and quality of life may improve with a supervised exercise program consisting of lower-extremity supine exercises.^3^

Although no drugs are currently approved by the United States Food and Drug Administration to treat POTS, fludrocortisone,^10,11^ pyridostigmine,^11,12^ low-dose propranolol,^13^ midodrine,^14^ droxidopa^15^ and β-blockers including bisoprolol^10,11^ may provide some benefit. Clonidine and methyldopa can be considered in neuropathic POTS.^3^ Recent trials have indicated a slight heart rate improvement with modest symptom improvement and minimal side effects following ivabradine administration.^16,17^ Acetylcholinesterase inhibitors represent another option.^18^ Additionally, administration of intravenous saline in hypovolemic patients has had some reported benefit and has been proposed to possibly prevent hospitalizations.^3^ Regular intravenous saline infusions, sinus node modification, and drugs blocking norepinephrine reuptake transporters are not recommended in POTS patients.^3^ Intravenous immunoglobulin is being explored^19^ for select patients with an immune form of POTS.

May et al.^1^ reported on patients with orthostatic hypotension and autonomic failure who drank 480 mL of tap water and subsequently acutely experienced an increase in blood pressure greater than 30 mmHg within five minutes of ingestion. This pressor response persisted for one hour. Healthy individuals were compared with these individuals without any appreciable response. This oral ingestion proved to elicit a greater response as compared with intravenous infusion. The proposed mechanism revolves around decreased systemic vascular resistance in patients with autonomic dysfunction. Interestingly, an increase in sympathetic activity and norepinephrine levels was noted with the ingestion of water; however, this effect was blunted by β-blockade. May et al.^1^ described this effect in nine patients with POTS syndrome who showed an appreciable drop in heart rate, although not one as exaggerated as that seen in our patient. Similar findings were described by Shannon et al. in a study of nine orthostatic-intolerant patients. After ingestion of room-temperature water, the previously seen tachycardic response was blunted slightly, with no appreciable change in seated blood pressure or heart rate. However, this change was also not as abrupt as that seen in the case we describe.^20^ Elsewhere, water and clear soup have been shown to improve orthostatic tolerance in POTS.^21^

In conclusion, we describe the use of water ingestion as an acute treatment of POTS in a 24-year-old female. In our clinic, she demonstrated a dramatic increase in her sinus rate as it nearly doubled upon standing. Her heart rate went back to her identical resting rate upon sitting. After ingestion of eight ounces (236.6 mL) of water and five minutes of rest, standing did not affect her heart rate. This phenomenon has rarely been described for POTS, and the actual mechanism remains poorly understood. Nevertheless, it should be considered a safe adjunct to treat patients with POTS. Given the difficulties inherent in treating POTS, acute water ingestion is a safe, quick, and easy intervention that can be performed in the outpatient setting. This phenomenon needs to be investigated further.

We present this case to spur discussion on the subject of water ingestion for the acute management of POTS. We additionally welcome further thoughts.
